# Retraction: The Effects of Matrix Metalloproteinase-9 on Dairy Goat Mastitis and Cell Survival of Goat Mammary Epithelial Cells

**DOI:** 10.1371/journal.pone.0345624

**Published:** 2026-03-24

**Authors:** The PLOS One Editors

After this article [1] was published, concerns were raised about Figs 3 and 4. Specifically, the following panels appear similar, despite reporting results from different organisms:

- In Fig 3B, when aspect ratio is altered:
  - The *S. aureus* panel of Fig 3B, the Ad-siGosB panel of Fig 7B in [2] and Panel 6 of Fig 2b in [3].
  - The SB-3CT (5 µM) panel of Fig 3B and Panel 7 of Figs 2b and 6b in [3].
- In Fig 4D, the Control Merge and Control EdU panels appear similar to the Fig 6D Ad-GosB + Ad-siGosB Merge and Ad-GosB + Ad-siGosB EdU panels in [2], when rotated 180 degrees.

The first author stated that the above concerns were the result of errors made during manuscript preparation. They provided underlying image and flow cytometry data for revised versions of Figs 3 and 4D, but not for the published figures, therefore the concerns remain unresolved. The underlying image data provided for the revised Fig 4D also raised further concerns with the reliability of these data. The original quantitative data underlying Figs 3 and 4 was not provided on editorial request.

In light of the above unresolved concerns which question the reliability of these data, the *PLOS One* Editors retract this article.

HL responded but expressed neither agreement nor disagreement with the editorial decision. HZ, LL, XS, WZ, and YS either did not respond directly or could not be reached.

The *S. aureus* and SB-3CT (5 µM) panels of Fig 3B report material similar to that published in [3], published in 2016 by Springer Nature, not under a CC BY license. The *S. aureus* and SB-3CT (5 µM) panels of Fig 3B are not offered under a CC BY license and are therefore excluded from this article’s [1] license.
